# Cardiovascular risk and inflammation in a population with autoimmune diseases: a narrative review

**DOI:** 10.3389/fimmu.2024.1380372

**Published:** 2024-03-28

**Authors:** Camilla Bertoni, Alessandra Mazzocchi, Ludovica Leone, Carlo Agostoni, Giovanni Filocamo

**Affiliations:** ^1^ Department of Veterinary Sciences for Health, Animal Production and Food Safety, University of Milan, Milan, Italy; ^2^ Department of Clinical Sciences and Community Health, University of Milan, Milan, Italy; ^3^ Pediatric Area, Fondazione Istituto di Ricovero e Cura a Carattere Scientifico (IRCCS) Ca’ Grande Ospedale Maggiore Policlinico, Milan, Italy

**Keywords:** dyslipidemia, inflammation, lipid profile, alpha-linolenic acid, autoimmune diseases

## Abstract

Juvenile Systemic Connective Tissue Diseases (JSCTD) are a heterogeneous group of chronic autoimmune diseases, associated with dyslipidemia and increased cardiovascular risk are related. Studies from the last 10 years, from 2013 to 2022, on lipid profiles in JSCTD were collected. Different studies on lipid profiles in children affected by JSCTD were selected, because the aim is to analyze the cardiovascular risk and the possibility of atherosclerosis in these patients in whom, sometimes, corticosteroid therapies and immunosuppressants increase the state of dyslipidemia. Several studies have shown that autoimmune diseases with an inflammatory substrate also share abnormalities in lipid profile and increased cardiovascular risk. Specifically, associations have been found between Juvenile Systemic Connective Tissue Diseases and elevated triglycerides, TC-C (Total Cholesterol), LDL-C (Low-Density Lipoprotein), low HDL-C (High-Density Lipoprotein), and increased risk of developing diseases such as myocardial infarction, peripheral vascular disease, pulmonary and arterial hypertension, and atrial fibrillation. Supplementation with alpha-linolenic acid (ALA) on the other hand has also been analyzed with positive results in reducing inflammatory parameters, such as IL-6 (Interleukin-6), CRP (C-reactive protein), and fasting glucose, in subjects with dyslipidemia. These observations suggest that supplementation with ALA, an omega-3 precursor, may positively modulate both the inflammatory status and dyslipidemic conditions in patients with autoimmune disorders.

## 1 Introduction

Juvenile Systemic Connective Tissue Diseases (JSCTD) are a heterogeneous group of juvenile-onset chronic autoimmune diseases, characterized by the presence of systemic inflammation and autoimmune-mediated multi-organ system involvement. The most common diseases in children are juvenile onset systemic lupus erythematosus (JSLE), juvenile dermatomyositis (JDM), juvenile systemic sclerosis (JSSC), juvenile localized scleroderma, juvenile mixed connective tissue disease (JMCTD) and juvenile Sjögren’s syndrome.

SLE is a chronic autoimmune disease characterized by autoantibody-associated multi-organ damage, including renal, cardiovascular, neural, and primarily affects women of childbearing age (1). Symptoms vary widely, from mild cutaneous manifestations to life-threatening organ failure (2). Childhood-onset SLE has a more aggressive disease course with greater morbidity and mortality than adult SLE (3).

JDM is a multisystemic disease of unknown etiology characterized by nonsuppurative inflammation of striated muscle and skin (4, 5). Most common forms of idiopathic inflammatory myopathy (IIM) are associated with marked activation and damage to small blood vessels in the connective tissues of the muscle and skin (6, 7). Capillary loss precedes other pathological muscle changes, and the inflammatory infiltrate in dermatomyositis is predominantly perivascular and perimysial (8). Vascular damage is considered integral to the disease pathogenesis of DM which also shows activated capillaries with increased adhesion molecule expression (9).

JSSC, also known as scleroderma, is a connective tissue disease (10). The etiology mirrors that of adult-onset systemic sclerosis (SSC), with similar inflammatory mediators and autoantibodies, but with a significant population of children with uncharacterized antinuclear antibodies (11). Several pathways in SSC vasculopathy can lead to atherosclerotic manifestations (12). SSC-related vasculopathy combined with immune dysregulation and progressive fibrosis are implicated in the pathogenesis of large vessel disease and specific cardiac dysfunction (13–15).

Sjögren’s syndrome is a systemic autoimmune disease that affects middle-aged women (16). Patients with this disease present with oral and ocular dryness, including interstitial pneumonitis, thyroiditis, central nervous system involvement and vasculitis (17). In the pediatric population, Sjögren’s syndrome is less characterized in terms of clinical presentation and long-term outcome (18).

## 2 Dyslipidemia, inflammation, and atherosclerosis in pediatric populations with autoimmune rheumatic diseases

Dyslipidemia has rarely been studied in pediatric populations with autoimmune rheumatic diseases. Lipid abnormalities in these diseases can occur due to various risk factors (body composition, corticosteroid therapy, chronic inflammation, etc) (19).

Dyslipidemia is diagnosed in 50-85% of children and adolescents with SLE (20–22), and is a major risk factor for heart failure and cardiovascular diseases (CVDs). The risk of CVD is twice as high in adults with SLE compared to the general population (23). Lipid metabolism affects both the innate and adaptive immune systems through various mechanisms, creating a vicious cycle that promotes atherosclerosis (22).

Studies in children and adolescents with SLE using biomarkers are still scarce in the literature (24–28). Several lipid abnormalities have been described in JSLE, including dyslipidemia with elevated triglyceride (TG) and LDL levels, decreased levels of HDL and apolipoprotein-A1 (Apo-A1), and an increased abundance of dysfunctional proinflammatory HDL, which lacks the antioxidant capacity of conventional cardioprotective HDL (23, 29–31).

Inflammation is a major driver of atherosclerotic disease. The process may be accelerated in JSLE patients due to the prolonged exposure to inflammation. Inflammation and atherosclerosis are causally related. A stringent lipid-lowering therapy is required to reduce the risk of adverse outcomes of atherosclerosis, and inflammation is currently considered a novel therapeutic target to counteract atherosclerotic complications (22). Inflammation is a fundamental pathological mechanism in autoimmune diseases. The cytokines released by the activation of the immune system can affect fatty acid oxidation, and activate lipoprotein lipase in adipose and muscle tissue, together with hepatic lipase, leading to dyslipoproteinemia (32). Dysregulation of lipid metabolism initiates an inflammatory and immune-mediated response in atherosclerosis (22).

However, the pathogenesis of dyslipidemia and accelerated atherosclerosis in SLE and JSLE has not been clearly identified (33). Several drugs commonly used in SLE patients may have adverse effects on lipid metabolism, such as corticosteroids (34), cyclosporine A (35), and tacrolimus (36). Metabolic disorders caused by a chronic excess of nutrients such as lipids and glucose can also accelerate the process too, and both conditions may simultaneously trigger pro-inflammatory responses (37, 38). Accordingly, an immune metabolic response may contribute to the abnormal lipid metabolism of SLE. Oxidized LDL, defective clearance of dead cells, and a pro-inflammatory T-cell profile are features of both atherosclerosis and SLE (2), underpinned by an imbalance in the immune system with lower levels of T-regulatory cells (Tregs) relevant for suppressing of autoimmune, pro-inflammatory responses against the self (39–41).

## 3 Dietary strategies on inflammatory markers

A very powerful nutrient that can help to reduce inflammatory markers is ALA, a precursor of the omega-3 series. ALA is one of the two essential fatty acids of plant origin and is the main precursor of eicosapentaenoic acid (EPA) and docosahexaenoic acid (DHA, which belong to the omega-3 series). ALA is mainly found in walnut oil, flaxseed oil, hemp oil, canola oil and seeds, and other types of vegetable oils. ALA reduces the risk of cardiovascular disease risk, possibly by favorably modifying vascular inflammation, with reduced levels of CRP, vascular cell adhesion molecule-1 (VCAM-1), E-selectin, serum total cholesterol, LDL and triglyceride levels. It may reduce CVD risk by inhibiting vascular inflammation and endothelial activation beyond its lipid-lowering effects (42). Dietary supplementation with ALA significantly reduced IL-6 levels (43). To support the cholesterol-lowering effect of ALA, EFSA (European Food Safety Authority) has endorsed a claim (44). In addition, ALA supplementation is also essential to maintain the omega-6/omega-3 ratio, which is currently unbalanced around 15/1 and 16/1 in the Western diet (45, 46). An imbalance of these nutrients in the diet, such as linoleic acid (LA), and a ratio of fatty acids clearly in favors of omega-6, favors the pathogenesis of many diseases, including cancer, inflammation and autoimmune diseases. On the other hand, a high intake of omega-3, especially ALA for example, suppresses inflammation. An excess of LA is therefore detrimental to omega-3 metabolism. Since omega-6 as LA is found in many more foods than ALA, it is easy to understand how easy it is to overdo LA intake and AA production (47).

## 4 Results

### 4.1 Dyslipidemia in JSLE

In the study by Machado et al. (48), adolescent females (n=33) aged 10-19 years old diagnosed with JSLE according to the American College of Rheumatology were evaluated (49). The control group consisted of 33 healthy adolescent females. Dyslipidemia was observed in 39.4% of the patients. Multivariate analysis showed that the SLE group had a lower ApoA1 concentration (163.1 vs 96.2, p value 0.001) and a lower LDL-C/ApoB ratio (1.1 vs 0.9, p value 0.001). As for the lipid profile, values of TC-C, TG and LDL-C levels were elevated in 9.1%, in 24.2% and 21.2% of SLE patients, respectively, while HDL-C concentrations were decreased in 24.2% of SLE patients.

Zhou et al. describe in the cross-sectional study of 2020 (50), the relationship between serum lipid profile and SLE disease activity in young female adults with SLE. Seventy-one female adults (20-30 years old) newly diagnosed with active SLE were included in this study. As expected, TG and VLDL levels were significantly elevated in the subjects with SLE and compared to controls (0.8 ± 0.03 vs 2.2 ± 0.06, p value < 0.01 for TG and 0.3 ± 0.02 vs 0.8 ± 0.03, p value < 0.01 for VLDL). HDL-C and ApoA1 were also significantly decreased in the SLE group compared to the control (1.6 ± 0.05 vs 0.8 ± 0.04, p value 0.02 for HDL-C and 1.3 ± 0.04 vs 0.9 ± 0.03, p value < 0.01 for Apo A). Levels of TC-C and LDL-C were decreased in the SLE group compared to the control group.

As for the pediatric and adolescent populations, the study by Ortiz et al. (51) evaluated the presence of dyslipidemia and cardiovascular risk factors, introducing homocysteine (Hcy) and cysteine (Cys) as additional markers of higher risk of developing CVD. A controlled cross-sectional study was conducted in 26 female adolescents with SLE and 26 with HC. Dyslipidemia was found in 46.2% of the patients. The SLE group had a lower HDL-C and higher Hcy and Cys concentrations than the HC group. In multivariate analysis only Hcy was significantly and independently associated with the presence of dyslipidemia in the JSLE group. The Cys concentration was correlated with Hcy, TC-C LDL-C, and TG concentrations. These data confirm the presence of cardiovascular risk factors in adolescents with JSLE.

A retrospective cross-sectional study of 2020 by Rodrigues et al. (52), aimed to describe the prevalence of dyslipidemia in children and adolescents with autoimmune diseases (ARDs), such as JSLE. They studied 186 children and adolescents between the ages of 5 and 19 years old and found dyslipidemia in 128 patients (68.8%), the most common being low HDL-C (74 patients, 39.8%). In JSLE patients, the cumulative corticosteroid dose was associated with an increase in LDL-C (p=0.013) and with a decrease in HDL-C (p=0.022).

### 4.2 Dyslipidemia in JDM

In a study by Kozu et al. (53), 33 JDM patients were enrolled from the Pediatric Rheumatology Unit of University Hospital. The patients had significantly higher levels of VLDL and TG compared to controls [16 (6–68) vs. 13 (4–36) mg/dL, p=0.02; and 80 (31–340) vs. 61 (19–182) mg/dL, p=0.011 respectively]. This group had a higher frequency of low HDL levels (28% vs 4%, p=0.004). In JDM, dyslipidemia was characterized by low HDL levels in seven patients (28%), high TG in four (16%) and high LDL in one (4%). These patients then had significantly higher levels of fasting glucose insulin levels [8 (3–45) vs. 3.9 (2–63) μU/ml, p=0.01], and lower fasting glycemia [80 (63-95) vs. 86 (76–102) mg/dL, p=0.009] and glucose/insulin rate [8.8 (1.5–32) vs. 19.7 (1.6–39), p=0.004] compared to controls.

Of particular interest is high-density lipoprotein function; Bae et al. (54) evaluated the antioxidant function of HDL in IIM patients in the 2020 study. Myositis patients and HC were recruited from the University of California, Los Angeles (UCLA). The cholesterol profile was measured in all patients, and subgroup analysis included assessment of oxidized fatty acids in HDL. The results showed that the antioxidant function of HDL was significantly worse in patients with IIM (n=95) compared to HC (n=41). These findings support the notion that the antioxidant function of HDL is abnormal in IIM patients and may warrant further investigation of its role in the propagation of microvascular inflammation and damage in this patient population.

### 4.3 Dyslipidemia in JSSC

A nationwide cohort study of 2019 by Butt et al. (55) included 2778 patients (76% female) with a diagnosis of SSC and 13.890 controls with a total follow-up of almost 9 years. Patients with SSC had more established cardiovascular risk factors than their respective controls at baseline, including a higher prevalence of hypertension (31.2% vs 21.0%, p<0.0001) and dyslipidemia, despite treatment (9.8% vs 8.5%, p=0.02). SSC was associated with an increased relative risk of developing most cardiovascular diseases, including myocardial infarction (HR: 2.1; 95% CI, 1.6–2.6), peripheral vascular disease (HR: 5.7; 95% CI, 4.6–7.1), pulmonary hypertension (HR: 21.9; 95% CI, 14.7–30.5), heart failure (HR: 2.9; 95% CI, 2.4–3.4), and atrial fibrillation (HR: 1.8; 95% CI, 1.5–2.0).

A study in 2022 by Zulian et al. (56) included 52 JSSC pediatric patients. Due to the lack of “pediatric” criteria for the diagnosis of this disease, the authors used adult criteria to select patients with JSSC. The results show that of these 52 patients, six had cardiac involvement, three had primary cardiomyopathy, two died after a short brief disease course and one underwent heart transplantation. JSSC patients had a significantly longer delay in diagnosis (20.1 vs 8.3 months, P=0.017), a higher frequency of cardiac involvement (85.7 vs 15.6%, P=0.001) and a worse outcome, defined as mortality or end-stage organ failure rates (42.9% vs 6.2%, P<0.001). Cardiac involvement and dyslipidemia are the main features of JSSC patients and are associated with high morbidity and mortality.

### 4.4 ALA: the anti-inflammatory effect

Among the nutraceuticals with anti-inflammatory effects, ALA is worth mentioning. Rallidis et al. (43) investigated whether dietary supplementation with ALA affects the levels of inflammatory markers in dyslipidemic patients. 76 male adults aged 71 years were assigned to receive 15 ml of linseed oil, rich in ALA per day. The dietary intervention lasted for 3 months. Blood lipids and CRP, serum amyloid A, and IL-6 levels were measured before and after supplementation. The results show a significant reduction in CRP (1.2 vs 0.9 mg/l; P=0.0008), serum amyloid A (3.2 vs 2.4 mg/l, P=0.0001) and IL-6 levels (2.2 vs 1.7 pg/ml, P=0.01). This anti-inflammatory effect may be a possible additional mechanism for the beneficial effect of plant omega-3 PUFAs in the primary and secondary prevention of coronary heart disease.

In a pilot study by Cavina et al. (57), the anti-inflammatory effect of ALA was evaluated in a group of 12 volunteers, aged 40-70 years with borderline dyslipidemia or overweight, who were treated with an ALA-based dietary supplement, according to international guidelines. The patients received 6 g/day of ALA for two months. Fasting blood glucose (FBS), CRP, transaminases (ALT, AST) were measured by routine methods. CRP levels in the supplementation group decreased from 0.8 ± 0.32 to 0.6 ± 0.19 (T=60); the HC group an increase in CRP concentrations, from 0.7 ± 1.4 to 0.7 ± 0.15. The results confirm that ALA is effective in reducing of CRP and it has an anti-inflammatory activity.

Niknam et al. (58) investigated associations of anti-inflammatory effects of dietary fatty acid intake. This cross-sectional study investigated the association between dietary fatty acid intake and inflammatory markers, such as IL-6, and high sensitivity C-reactive protein (hs-CRP) in patients with coronary artery disease (CAD). The study included 150 CAD male patients aged ≥ 45. Participants were asked to report how often they had eaten a certain amount of each food item on a daily, weekly, or monthly basis in the past year. The effect of fatty acid intake, including saturated fat, monounsaturated fat, LA, ALA, EPA, and DHA on plasma levels of inflammatory markers was then measured. Partial correlation analysis was performed to evaluate the association of fatty acid intake with plasma IL-6 and hs-CRP concentrations. The correlation between EPA+DHA and IL-6 was -0.3, p=0.003 and hs-CRP was -0.4, p<0.001. Monounsaturated fat was significantly inversely related to plasma levels of IL-6 and hs-CRP, whereas SFA was directly related to IL-6 and hs-CRP. There was a significant association between ALA, IL-6, and hs-CRP. No significant associations were found for LA and IL-6 and hs-CRP. The results of this study suggest an inverse relationship between omega-3 fatty acids, EPA+DHA, and MUFA (monounsaturated fatty acids) with hs-CRP concentration in CAD patients. SFA intake was directly related to IL-6 and hs-CRP levels.

## 5 Discussions

Cardiovascular disease is the leading cause of death in the most developed countries, accounting for about 30% of all deaths (59). Research is trying to find ways to act directly on risk factors such as atherosclerosis, hypertension, dyslipidemia including hypercholesterolemia and hypertriglyceridemia, chronic and systemic inflammation, and insulin resistance. The first confirmed effect of omega-3 is its cholesterol-lowering action, as well as its anti-inflammatory, anti-thrombotic, anti-atherosclerotic, and anti-arrhythmogenic functions. In fact, it has been observed that a situation of progressive inflammation is closely linked to an increased cardiovascular risk, which can manifest itself as atherosclerosis or coronary heart disease (60, 61).

There is a close correlation between lipid profile and plasma membrane composition in terms of fatty acids. Both omega-3 and omega-6 produce eicosanoids, molecules that regulate inflammation. Eicosanoids play an important role in eliminating systemic inflammation. To explain the existing link between cardiovascular risk and inflammation, studies have shown that an increased intake of fatty acids that are deposited at the level of the plasma membrane is an important factor in reducing systemic inflammation and, consequently, cardiovascular risk (62).

Studies conducted over the last 20 years have demonstrated the efficacy and usefulness of these nutrients significantly reducing LDL and total cholesterol levels, improving erythrocyte membrane composition in terms of PUFA, and minimizing inflammation. There are important correlations, linking increased omega-3 intake to reduced inflammation, manifested by changes in eicosanoid metabolism, and reduced cytokine secretion. Significant reductions in TNF-α, IL-6, and inflammatory markers, including hs-CRP, are seen when are administered to reduce systemic inflammation. In this context, low-grade inflammation plays a key role in the progression of atherosclerosis and consequently increases cardiovascular risk. Therefore, the use of ALA, as opposed to longer chain derived omega-3 compounds, may be critical in achieving cardiovascular risk reduction due to its antioxidant, anti-inflammatory and anti-arrhythmic effects (62).

## Author contributions

CB: Writing – original draft, Writing – review & editing. AM: Writing – original draft, Writing – review & editing. LL: Writing – original draft, Writing – review & editing. CA: Writing – original draft, Writing – review & editing. GF: Writing – original draft, Writing – review & editing.
